# Molecular Characterization of katG and inhA Mutations by Genotype MTBDRplus Line Probe Assay To Guide Isoniazid and Ethionamide Use for Drug-Resistant Tuberculosis

**DOI:** 10.7759/cureus.37136

**Published:** 2023-04-04

**Authors:** K. P. Ranjan, Neelima Ranjan, Nitin Kumar

**Affiliations:** 1 Department of Microbiology, Gajra Raja Medical College, Gwalior, IND

**Keywords:** cross resistance, drug resistant tuberculosis, line probe assay, genotype mtbdrplus assay, isoniazid resistance

## Abstract

Introduction

Drug-resistant tuberculosis (TB) continues to be a global health threat in all its forms. Significant resistance has been observed against isoniazid (INH), one of the most important therapeutic options for treating TB. Molecular testing methods such as line probe assay (LPA) provide rapid diagnosis and early management. Mutations in different genes can be detected, which indicate INH and ethionamide (ETH) drug resistance. We aimed to determine the frequency of these mutations in katG and inhA genes by LPA to guide INH and ETH use for drug-resistant TB.

Materials and methods

Two consecutive sputum samples were collected from each patient, followed by decontamination by N‑acetyl‑L‑cysteine and sodium hydroxide method. LPA was performed on the decontaminated samples by GenoType MTBDR*plus*, and the strips were analyzed.

Results

Out of the 3,398 smear-positive samples tested by LPA, valid results were found in 3,085 (90.79%) samples. Of the 3,085 samples, INH resistance was seen in 295 samples (9.56%), of which mono INH resistance was in 204 samples, and 91 were multidrug resistant. katG S315T was the most common mutation responsible for high-level INH resistance. At the same time, inhA c15t was the most common mutation associated with low-level INH resistance and ETH cross-resistance. The average turnaround time for the processing and reporting of samples was five days.

Conclusions

The high burden of INH resistance is alarming and can be a major obstacle to TB elimination. Although molecular methods have reduced the reporting time leading to early management of the patients still, a large knowledge gap persists.

## Introduction

Tuberculosis (TB) remains a major cause of ill health and one of the leading causes of death worldwide by a communicable disease. Until the COVID-19 pandemic, TB was the leading cause of death from a single infectious agent, ranking above HIV infection. As per the Global TB Report 2021, the estimated incidence of all forms of TB in India for the year 2020 was 188 per 100,000 population (129-257 per 100,000 population) [1].

The introduction of molecular methods has revolutionized TB diagnostics, especially the rapid determination of susceptibility and resistance to anti-TB drugs. These methods have been seen to have equivalent accuracy and are known to significantly reduce the turnaround time compared to conventional culture methods [2]. In 2008, the WHO endorsed the use of the first line (FL) line probe assay (LPA), GenoType MTBDRplus, for the rapid detection of multi-drug resistant tuberculosis (MDR-TB) [3]. LPA is a rapid technique based on polymerase chain reaction (PCR) and reverse hybridization that is used to detect mutations associated with the rpoB gene for rifampicin resistance, katG gene and the inhA regulatory region gene for high‑level isoniazid (INH) resistance and low‑level INH resistance, respectively [4].

Rifampicin (RIF) and INH are the pillars of treatment for drug-sensitive TB. INH is a very effective drug as it exhibits early mycobactericidal activity by inhibiting mycolic acid biosynthesis, which is an integral part of the Mycobacterium tuberculosis (*M. tuberculosis*) cell wall [5]. Across the world, the INH mono-resistance in new and previously treated cases was observed to be 7.2% and 11.6%, respectively. In India, INH mono-resistance was observed in 3.8% and 7.8% of new and treated patients, respectively [6]. Previous researches suggest that if unidentified or mistreated, INH mono-resistance may increase the chances of progression to MDR-TB and poorer treatment outcomes [7]. INH resistance commonly occurs due to mutations in the katG gene or the inhA regulatory regions. katG codes for catalase-peroxidase, which is an enzyme that converts INH to its biologically active form. As mutations in katG, particularly at codon 315, confer high-level INH resistance, INH, even at high doses, is ineffective for treating *M. tuberculosis* with this mutation profile [8]. The inhA regulatory region encodes nicotinamide adenine dinucleotide-dependent enoyl-acyl carrier protein reductase, the primary target of active INH, and ethionamide (ETH) and prothionamide (PTH). inhA mutations cause low-level resistance to the drug, which means that high doses of INH may be effective against *M. tuberculosis*. ETH is a structural analog of INH, so cross-resistance occurs between INH and ETH. Low-level resistance to INH (inhA mutated) can also confer resistance to ETH, while *M. tuberculosis* with high-level INH resistance (katG mutated) is susceptible to ETH [9].

LPA has been used as a rapid means of detecting inhA mutation and associated phenotypic ETH resistance. The detection of mutation in inhA using rapid molecular tests such as LPA can help clinicians decide whether the patient will benefit from using high-dose INH or whether to prescribe ETH for the treatment of drug-resistant tuberculosis (DR‑TB). The less turnaround time (five days) with this test will also ensure timely treatment, which is beneficial to the patient and reduces the transmission of the infection [10]. The prevalence of such gene mutations has been seen to vary in different geographical areas. So, identifying these mutations may help to understand the epidemiology of the disease and also guide clinicians in better decision-making for the treatment of DR-TB patients. In this study, we aimed to determine the frequency of mutations in katG and inhA genes by LPA in Gwalior and another fourteen neighboring districts of Madhya Pradesh, India.

## Materials and methods

This retrospective study was conducted in the Tuberculosis Culture and DST laboratory (National TB Elimination Programme Certified Laboratory), Department of Microbiology, Gajra Raja Medical College, Gwalior, Madhya Pradesh, India. Institutional Ethical Committee at Gajra Raja Medical College, Gwalior, Madhya Pradesh, India, has approved the study (IRB No.: 160/IEC-GRMC/2022). Sputum samples from suspected TB patients from the out and inpatient departments of the J. A. Group of Hospitals, Gwalior, and 14 other neighboring districts of Madhya Pradesh from January 2021 to December 2021 were processed. All samples analyzed were collected as part of routine diagnosis and treatment. The diagnostic protocol we evaluated was the national protocol per Programmatic Management of Drug Resistant TB (PMDT) guidelines [11]. 

Specimen collection and processing

A total of 5,803 sputum samples from TB suspects were received and decontaminated by using N‑acetyl‑L‑cysteine and sodium hydroxide method in a class II biosafety cabinet [12]. All specimens were subjected to smear preparation, Ziehl‑Neelsen staining, and microscopy. A total of 3,398 (58.55%) and 2,405 (41.44%) specimens were found smear-positive and smear-negative, respectively. Out of the total smear-positive samples, 993, 643, 998, and 764 samples were 3+, 2+, 1+, and scanty positive, respectively, as per National Tuberculosis Elimination Programme (NTEP) guidelines [11]. All the smear-positive samples were centrifuged, and sediments were suspended in 2 ml sterile phosphate buffer for processing by GenoType MTBDRplus assay.

Genotype MTBDRplus assay

The GenoType MTBDRplus assay (Hain Lifescience, Nehren, Germany) is based on DNA strip technology. It has three steps: DNA extraction, multiplex PCR amplification, and reverse hybridization. These steps were carried out in three separate rooms with restricted access and unidirectional workflow. DNA was extracted from decontaminated samples using the Genolyse kit as per the manufacturer’s instructions. All reagents needed for amplification are included in amplification Mix A and B. For PCR, 10 µL of Mix-A and 35 µL of Mix-B were added and properly mixed with each other. Then, 5 µL of MTB DNA (extracted from sputum-positive samples) was added to the master mix, making the final volume of 50 µL. Multiplex PCR (Applied Biosystems) was performed using a thermocycler and amplification protocol provided by Hain Lifescience [13]. For hybridization, 20 µL of the amplification products and 20 µL of the denaturing reagent (provided with the kit) were mixed and allowed to stand for five minutes at room temperature. This mixture was then treated with hybridization buffer followed by stringent buffer and conjugate. The streptavidin-conjugated alkaline phosphatase bound to the amplicon, and finally, bands were visible after adding substrate. After completion of hybridization, strips were washed, removed, and fixed to the GenoType MTBDRplus assay worksheet for interpretation [13]. The standard strain, H37Rv, was used as a positive control, and molecular-grade water was used as a negative control in each batch [13].

Interpretation of genotype MTBDRplus kit

**Figure 1:**
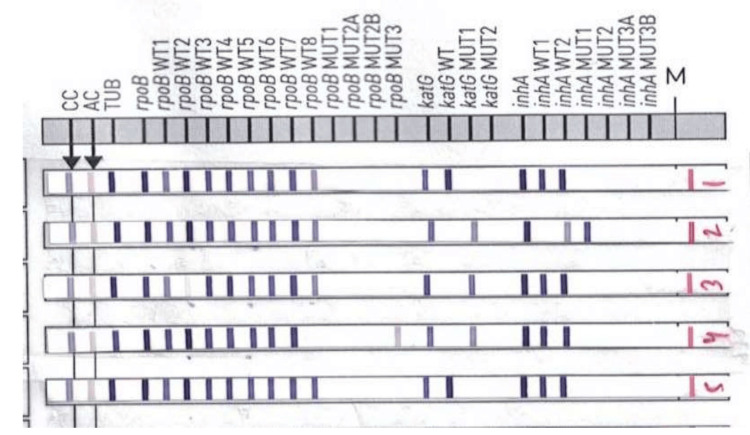
Rifampicin and isoniazid drug-resistant detection by GenoType MTBDRplus. Lane 1. Susceptible to Rifampicin and Isoniazid Lane 2. Mono-isoniazid resistant detected with katG S315T1 and inhA C15T mutation Lane 3. MDR with rpoB 510-513 gene region mutation and katG S315T1 mutation Lane 4. MDR with rpoB S531L mutation and katG S315T1 mutation Lane 5. Susceptible to Rifampicin and Isoniazid MDR: Multi-drug resistant.

Each strip had 27 reaction bands (LPA strip shown in Figure 1), including three controls: conjugate control (CC), amplification control (AC), M. tuberculosis complex (TUB), and three locus controls: rpoB locus, katG locus, and inhA locus. The remaining 21 reaction zones were of wild type (WT) and mutation reaction zones, including eight rpoB WT (WT1-WT8), four MUT probes (rpoB MUT D516V, rpoB MUT H526Y, rpoB MUT H526D, and rpoB MUT S531L), one katG WT, two mutant probes (katG MUT S315T1 and katG MUT S315T2), two inhA WT, and four mutant probes (inhA MUT C15T, inhA MUT A16G, inhA MUT T8C and inhA MUT T8A). Results were interpreted according to the manufacturer’s instructions [13]. All six expected control bands should appear on a strip to give a valid result. Otherwise, the result was considered invalid. In a valid test, when all the WT bands were present, and no mutation band was present, the isolate was reported as Resistance Not Detected. The absence of the WT band is usually accompanied by the presence of a mutation band, which indicated resistance and was reported as Resistance Detected. The detection of mutation and the WT bands was considered heteroresistance, while the absence of mutation and WT bands was considered inferred resistance [14]. Turnaround time (TAT) was calculated from the collection of samples to the availability of online results. Isolates showing resistance to rifampicin alone were not included in the study as we were focusing on INH resistance in this study. Data were presented as frequency tables, and mean and percentage were calculated wherever required.

## Results

**Figure 2:**
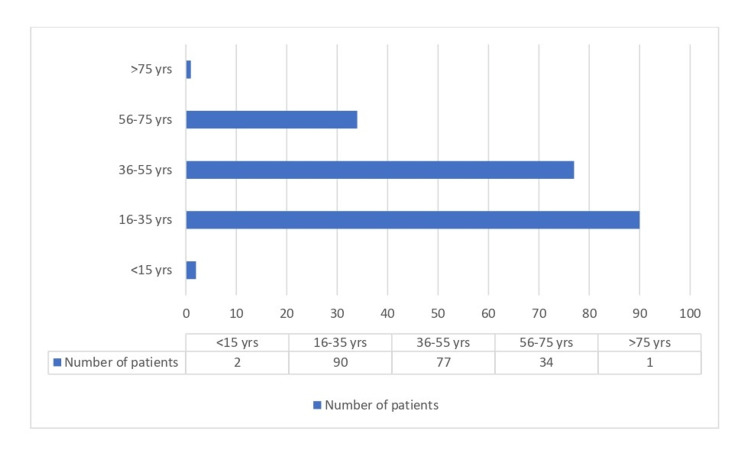
Age-wise distribution of mono INH-resistant samples. INH: Isoniazid.

**Figure 3:**
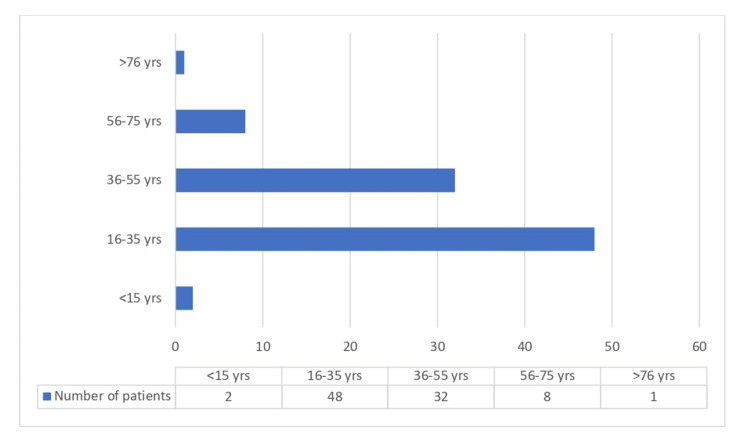
Age-wise distribution of MDR samples. MDR: Multi-drug resistant.

A total of 3,398 smear-positive samples were tested by LPA. Valid LPA results were found in 3,085 (90.79%) samples, and 313 (9.21%) samples were found to be negative for the MTB band on the LPA strip. Of the 3,085, INH resistance was seen in 295 samples (Table 1). Out of these, 71.5% of patients were males, and 28.6% were females, the ratio of males to females being 2.5:1. The maximum number (47%) of patients were from the age group 16-35 years. Of the 295, 211 and 84 samples were from new and previously treated patients, respectively. The detailed distribution of samples according to sex, age, and history of previous antitubercular treatment is shown in Table 2 and Figures 2-3. Of the 3,085 samples, 204 (6.61%) were resistant to INH only, and in 91 (2.94%) samples, resistance was detected for both RIF and INH. When considering the individual mutations, 134 (65.68%) samples showed resistance in katG, 68 (33.33%) in inhA, and 2 (0.98 %) samples had mutations in both (katG and inhA). The average TAT from receiving samples in the laboratory to reporting on the online portal was five days.

**Table 1:** Distribution of mono INH resistant and MDR samples (out of 3,085 samples). INH: Isoniazid; MDR: Multi-drug resistant.

Description	Number of samples
Mono-INH resistant	204 (6.61 %)
RiIF + INH resistant	91 (2.95 %)
Total	295 (9.56 %)

**Table 2:** Sex-wise distribution of mono INH and INH+RIF resistant samples. INH: Isoniazid; RIF: Rifampicin.

Description	Mono-INH resistant (204)		Rif + INH resistant (91)		Total samples (295)
	Male	Female	Male	Female	
Newly diagnosed patients	105	41	45	20	211
Previously treated patients	41	17	20	6	84

Molecular characterization of drug-resistant samples

In mono INH-resistant samples, missing katG WT1 accompanied by the presence of a mutation at S315T1 depicting high-level INH resistance was found in 113 out of 204 samples. Low-level INH resistance and ethionamide/prothionamide cross-resistance were seen in 51 samples showing absent inhA WT1 and the presence of inhA MUT1, most of which could be accounted for due to c15t mutations. Twenty-seven samples showed a heteroresistance pattern (WT and MUT bands were present simultaneously), with 16 samples showing heteroresistance in katG and 11 in inhA. Inferred band patterns were seen in six samples, while both katG and inhA MUT bands were seen in two samples. The mutation profile in mono-INH-resistant samples is shown in Table 3.

**Table 3:** Mutation profile of katG and inhA in mono-INH-resistant samples. INH: Isoniazid.

Description	Absent WT band	Codon analysed	MUT band developed	Mutation	Number of samples/Percentage
Resistance detected	Kat G WT	315	Kat G MUT1, Kat G MUT2	S315T1, S315T2	113 (55.4), 2 (1)
Resistance inferred	Kat G WT	315	_	_	03 (1.5)
Heteroresistance	_	315	Kat G MUT1	S315T1	16 (7.7)
Resistance detected	Inh A WT1	15 16	Inh A MUT1, Inh A MUT2	c15t, a16g	51 (25), 01 (0.5)
	Inh A WT2	16 8	Inh A MUT2, Inh A MUT3A	a16g, t8c	1 (0.5), 1 (0.5)
Resistance inferred	Inh A WT1	-	-	-	3 (1.5)
Heteroresistance	_	15 8	Inh A MUT1, Inh A MUT3B	c15t, t8a	10 (5), 01 (0.5)
Resistance detected	Kat G WT+ inh A WT1	315, 15	Kat G MUT1 + inh A MUT1	S315T1, c15t	2 (1)

In MDR cases, most samples exhibited katG mutation, and only three samples exhibited mutation in the inhA gene alone. Ten samples had mutation bands in both katG and inhA. Heteroresistance and inferred band patterns were noted in the katG gene only in six and two samples, respectively. The detailed mutation pattern of these MDR isolates is shown in Table 4. 

**Table 4:** Mutation profile of katG and inhA in MDR samples. MDR: Multi-drug resistant.

Description	Absent WT band	Codon analyzed	MUT band developed	Mutation	Number of samples/Percentage
Resistance detected	Kat G WT	315	Kat G MUT1, Kat G MUT2	S315T1 ,S315T2	69 (75.8), 1 (1.1)
Resistance inferred	Kat G WT	315	_	_	2 (2.2)
Heteroresistance	_	315	Kat G MUT1	S315T1	6 (6.6)
Resistance detected	Inh A WT1	15	Inh A MUT1	c15t	3 (3.3)
Resistance inferred	_	_	_	_	_
Heteroresistance	_	_	_	_	_
Resistance detected	Kat G WT+ inh A WT1	315, 15	Kat G MUT1+ inh A MUT1	S315T1, c15t	8 (8.8)
	Kat G WT + inhA WT2	315, 8	Kat G MUT1+ inh A MUT3A	S315T1, t8c	2 (2.2)

## Discussion

Drug-resistant TB threatens global TB control measures and can make the elimination of TB by 2025 very difficult. Conventional phenotypic TB drug susceptibility testing (DST) is time-consuming. Resistant TB can be transmitted to more people as first-line TB medications may delay appropriate treatment and, consequently, sterilization of the sputum. Rapid molecular techniques like LPA have decreased the TAT drastically, allowing for quick initiation of correct treatment. In this study, test results for resistance to INH and RIF had an average TAT of five days, as compared to culture techniques which can take average 6-12 weeks. INH is the backbone of drug-sensitive TB treatment and an essential constituent of shorter-course MDR TB treatment. INH mono-resistance forms a major chunk of drug-resistant TB. In our study, resistance to RIF and INH was observed to be 9.6%, while INH monoresistance was found in 6.6% of samples, consistent with previous Indian studies [15-17]. The highest resistance to INH was seen in the age groups of 16-35 years. A higher positivity rate was seen in males, which has also been noted in previous studies [15]. This emergence of INH mono-resistance can be attributed to many factors, like previous tuberculosis treatment, HIV co-infection, and the confounding effect of INH preventive therapy [18,19]. Although INH resistance is associated with resistance-conferring mutations in multiple genes, katG and inhA account for ~90% of resistance detected by phenotypic DST. INH resistance due to katG gene mutation was documented in 71.9% of INH-resistant study samples, which agrees with previous studies [15,20]. S315T1 mutation was the most common (96.2%) mutation responsible for high-level INH resistance. This was also the only mutation in the samples showing a heteroresistance pattern in the katG gene. DR-TB patients exhibiting high-level INH resistance due to the katG mutation are unlikely to benefit from high-dose INH in the bedaquiline (BDQ) containing modified MDR-TB short regimen. However, they may benefit from the use of ETH [9]. Low-level INH resistance with cross-resistance for ETH/prothionamide resistance was found in 24.07% of INH-resistant isolates. Mutations in the inhA promoter, with c15t being the most prevalent mutation in our study, tend to be associated with low-level INH resistance. These patients might benefit from treatment with high doses of INH, but ETH may not be effective. The simultaneous presence of both katG and inhA mutations (4.06% in our study) is unlikely to respond to either high-dose INH or ETH. These patients will require alternate TB treatment regimens [9].

In eight samples, there was an absence of hybridization to the WT probe without the development of any ­mutation band. Such mutations are termed ‘inferred’ mutations as defined in the latest Global Laboratory Initiative guidelines (GLI, 2018) [14]. The reason for these ‘inferred’ mutations could be the presence of rare/novel/silent mutations, which are not included as MUT probes in LPA. Heteroresistance mutation patterns were seen in 11.2% of samples in our study. Early initiation of an MDR regimen among patients harboring heteroresistant isolates in comparison to MDR-TB isolates has been observed to have better treatment outcomes [16]. Initial INH resistance increases incidence rates of treatment failure and relapse compared with pan-sensitive strains [21]. One of the limitations of our study is the retrospective design which might have introduced bias in the selection of patients. Also, we did not have comparative phenotypic DST to correlate the performance and agreement between genotypic and phenotypic DST. Evaluation of the prevalence of ahpC, ethA, and the ethR mutations, in addition to inhA, is needed to better understand the resistance against INH and ETH. As long as these mutations are not included in LPA, we will have to rely on sequencing-based methods to understand the whole picture.

## Conclusions

INH and ETH are integral drugs for tuberculosis control. The high burden of INH mono-resistance (6.6%) and low-level INH resistance with cross-resistance to ETH, as seen in this study, suggests that the use of these mutation tests will be essential to guide early appropriate TB treatment. Further TB drug mutation studies are urgently required to detect the full scope of these resistance patterns in hopes of decreasing transmission and thereby helping fulfill the vision of TB eradication.
